# Modulation of the autonomic nervous system by one session of spinal low-level laser therapy in patients with chronic colonic motility dysfunction

**DOI:** 10.3389/fnins.2022.882602

**Published:** 2022-09-01

**Authors:** M. Khawar Ali, Shrayasee Saha, Natalija Milkova, Lijun Liu, Kartik Sharma, Jan D. Huizinga, Ji-Hong Chen

**Affiliations:** ^1^Faculty of Engineering, School of Biomedical Engineering, McMaster University, Hamilton, ON, Canada; ^2^Division of Gastroenterology, Department of Medicine, Faculty of Health Sciences, Farncombe Family Digestive Health Research Institute, Hamilton, ON, Canada

**Keywords:** autonomic nervous system, low level laser (light) therapy (LLLT), constipation, respiratory sinus arrhythmia, Baevsky stress index, sacral neuromodulation

## Abstract

Patients with a defecation disorder may not evoke a normal defecation reflex, or the reflex may be excessive, as a dysfunction of the spinal autonomic nervous system. Treatment with various forms of lumbar and sacral neuromodulation have shown symptom improvement, but potential changes in autonomic functioning are rarely studied. Here we evaluate the effects on autonomic function of a single session of low-level laser therapy (LLLT) on the lumbar and sacral spine in 41 patients with chronic gastrointestinal motor dysfunction. The LLLT protocol used red LED light at a wavelength of 660 nm for 10 min and infrared LED light at a wavelength of 840 nm for 10 min, followed by infrared laser light at a wavelength of 825 nm for 10 min. Effects on the autonomic nervous system were assessed by measuring heart rate variability (HRV) changes. Respiratory Sinus Arrhythmia (RSA) and Root Mean Square of Successive Differences (RMSSD) were used to quantify parasympathetic reactivity; the Baevsky’s Stress Index (SI) reflected sympathetic activity while the ratios SI/RSA and SI/RMSSD were used to show shifts in autonomic dominance. The results indicate that lumbar and sacral neuromodulation using light arrays reduced, whereas stimulation by the laser probes significantly increased parasympathetic activity. The light arrays increased whereas the laser probes significantly decreased sympathetic activity (SI). The entire protocol shifted the autonomic balance toward parasympathetic activity. The comparison of actual vs. sham neuromodulation proved that the change in HRV parameters was due to actual light stimulation and not due to the arrays and probe touching the skin. In conclusion, a single session of LLLT markedly affects autonomic nervous system activity reflected in changes in HRV which is only possible by generating activity in the spinal autonomic nerves. These results warrant a study into the effects of LLLT on restoring autonomic dysfunction in chronic refractory colonic motility disorders.

## Introduction

Sacral neuromodulation is explored as a treatment for motor disorders of the gastrointestinal and urinary tracts (Jones et al., 2016; Goldman et al., 2018; Payne et al., 2019). Short term stimulation of sacral nerves with implanted electrodes decreased fecal incontinence (Vaizey et al., 1999) and a multicenter study showed that chronic stimulation of the spinal cord with implanted electrodes in patients with fecal incontinence is a safe and effective treatment (Wexner et al., 2010).

Neuromodulation for chronic constipation has been explored to a lesser extent compared to fecal incontinence. In a prospective study at 5 European sites, 39 out of 45 patients achieved improvement with stimulation of implanted electrodes, from 2.3 to 6.6 evacuations per week (Kamm et al., 2010). Patients with chronic constipation associated with neurological disease also showed improvement (Khan et al., 2014). In other studies, success for alleviation of constipation was more limited (Thaha et al., 2015; Widmann et al., 2019).

The inference from many studies on chronic stimulation with implanted electrodes is that the sustained effects are caused by neuromodulation in response to repeated stimulation of both sensory and efferent fibers, causing changes in organ function. Kenefick et al. (2003) studying laser Doppler rectal mucosal blood flow, showed that activation of implanted sacral electrodes caused a marked increase in blood flux within seconds, suggesting modulation by extrinsic neural activity. Stimulation with implanted electrodes was also shown to create an electromyography response and contraction of the external anal sphincter *via* a polysynaptic reflex (Fowler et al., 2000). Furthermore, it evokes responses in the sensorimotor learning centers in cortical and subcortical structures, assessed by MRI (Blok et al., 2006).

Sacral neuromodulation using non-invasive methods is also pursued (Chen et al., 2017). Transcutaneous electrical nerve stimulation (TENS) has been shown to improve constipation symptoms in children (Leong et al., 2011; Veiga et al., 2013, 2016; Kim and Yi, 2014). Transabdominal electrical stimulation has also shown promise for lasting improvement of constipation (Leong et al., 2011). A systematic review showed that TENS had a significantly larger effect on stool frequency compared to placebo (Zheng et al., 2019). In rats, it was shown that TENS improved constipation *via* modulation of the autonomic nervous system, increased vagal activity and decreased sympathetic activity, assessed by spectral analysis of heart rate variability (HRV) (Huang et al., 2019).

Neuromodulation can also be achieved by low-level laser therapy (LLLT) or photobiomodulation. LLLT has a photochemical effect, where the application of specific frequencies of light causes chemical changes in the tissue. There is ongoing research about the cellular and molecular mechanisms through which LLLT promotes healing. It is well established that LLLT improves wound healing and reduces pain and inflammation (Mester et al., 1976). Activation of photoreceptor molecules inside the mitochondria results in increased adenosine triphosphate and reactive oxygen species, followed by activation of transcription factors producing anti-apoptotic, anti-oxidant, and pro-proliferation gene products (Hashmi et al., 2010; Chung et al., 2012). Increased ATP production from LLLT also upregulates the production of nitric oxide, which is a potent vasodilator and allows for increased blood flow and, therefore, nutrient delivery to the areas being stimulated (Hashmi et al., 2010; Chung et al., 2012). LLLT was able to enhance neural regeneration in rats following chronic depression of dorsal root ganglia (DRG) and improve their ambulatory behavior (Chen et al., 2014); the neuro-reparative effect through photobiomodulation has thus far been proven in painful diabetic neuropathy and various other neurological conditions (Hashmi et al., 2010; Yamany and Sayed, 2012; Rola et al., 2014; Chen et al., 2014; Andreo et al., 2017).

Our overall objective is to develop new treatments for patients with severe chronic colonic motility disorders, refractory to established treatments (Rao et al., 2016; Camilleri et al., 2017; Chen and Huizinga, 2019). Our specific objective related to neuromodulation is to evaluate if sacral neuromodulation can relieve symptoms and restore autonomic dysfunction in these patients (Liu et al., 2022). Patients with chronic constipation may not be able to generate spontaneous bowel movements. Constipation is the inability to generate one or more defecation reflexes that are orchestrated by the extrinsic autonomic nervous system involving propulsive motor activity and anal sphincter relaxation (Bharucha et al., 1993; Rao et al., 2016; Camilleri et al., 2017; Callaghan et al., 2018; Milkova et al., 2020; Ali et al., 2021). A propulsive colonic motor pattern may start with triggering of afferent neurons whose cell bodies lie within the DRG of the lumbar and sacral portions of the spinal cord (Brookes et al., 2009). Then, sacral parasympathetic nerves may initiate motor patterns in the descending colon, stimulate the rectum, and relax the internal anal sphincter in preparation for defecation (Brookes et al., 2009; Huizinga et al., 2020, 2021). At the same time, sacral information projects to the Barrington’s nucleus and the nucleus tractus solitarius through spinal pathways (Valentino et al., 1999). Barrington’s nucleus can then project the information to the vagus nerve through the dorsal motor nucleus of the vagus. The vagus nerve can invoke propulsive motor patterns in the ascending and transverse parts of the colon, thus transporting more colonic content in the anal direction (Valentino et al., 1999; Brookes et al., 2009). Through the neural activity in the brain stem, particularly the NTS, the neural traffic originating in the sacrum can influence autonomic innervation to the heart and hence affect HRV (Yuan et al., 2020; Ali et al., 2021).

HRV analysis is explored to evaluate autonomic functioning and dysfunction (Shields, 2009; Huizinga et al., 2018; Barbier et al., 2022; Liu et al., 2022). We used HRV successfully to show that propulsive motor patterns generated by the human colon are associated with an increase in parasympathetic activity and a decrease in sympathetic activity (Yuan et al., 2020; Ali et al., 2021). HRV is also used to assess success of treatment of autonomic dysfunction in animal models (Ouyang et al., 2020) and human studies (Karpyak et al., 2004; Euteneuer et al., 2022).

The goal of neuromodulation of the spinal cord is to affect the neural circuitries that are underlying the defecation reflexes; to drive them into their homeostatic state. It was, therefore, important to investigate whether or not LLLT can trigger autonomic nerves. The aim of the present study was to examine whether one treatment session of LLLT would show autonomic reactivity, assessed *via* HRV (Thayer et al., 2012; Baevsky and Chernikova, 2017; Ali et al., 2021), which would prove its ability to activate autonomic spinal nerves.

## Materials and methods

### Participants

Forty-one patients (male = 13, female = 28, age = 37 ± 17 years) with colonic motility disorders took part in this study. Out of 41 patients, 28 had chronic constipation, 5 had fecal incontinence while 8 were suffering from both constipation and fecal incontinence. The study was carried out with ethics approval from the Hamilton Integrated Research Ethics Board and written consent was obtained from all participants. All participants underwent concurrently one session of LLLT, ECG and impedance recording. Six healthy volunteers without any motility and cardiac disorders took part in a sham study.

### Heart rate and impedance measurements

ECG signals were recorded using MindWare BioLab Recording Software. MindWare HRV 3.1 software was used for artifact correction and to calculate the values of the beat-to-beat intervals (RR intervals), respiratory sinus arrhythmia (RSA), and root mean square of successive differences (RMSSD) (Thayer et al., 2012; Ali et al., 2021). The sampling frequency was 500 Hz. Baevsky’s stress index (Baevsky and Chernikova, 2017) (SI) was calculated using code developed in MATLAB using the RR interval time series. RSA and RMSSD were used as measures of parasympathetic reactivity; SI was used as a measure of sympathetic reactivity. The ratios SI/RSA and SI/RMSSD were used to measure shifts in combined parasympathetic and sympathetic activity. The HRV parameters were calculated for 6 min (baseline) before the LLLT session, during the three stages of the LLLT protocol, as well as 6 min recovery after the one time LLLT session. HRV parameters are obtained by short or 24 h monitoring periods; a 6 min period appears optimal for analysis of short intervention protocols (Shaffer and Ginsberg, 2017).

### Low-level laser therapy protocol

**FIGURE 1:**
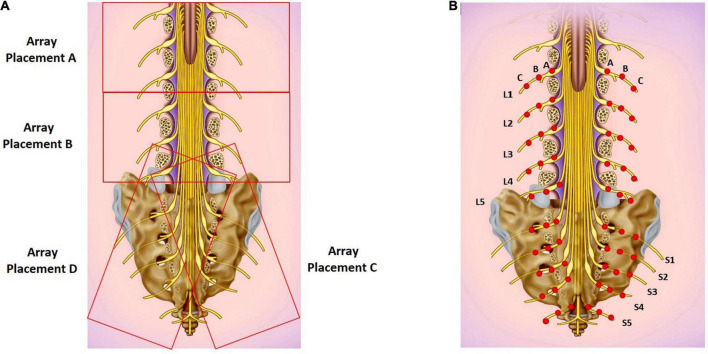
**(A)** LED array placements A, B, C, and D. **(B)** Target areas for laser probe stimulation marked as red dots. Each point is stimulated for 20 seconds. Basic image obtained from dreamstime.com with permission.

Two BioFlex Duo + Professional Systems were used simultaneously to provide light therapy to the lower back; each BioFlex Duo + system included a control unit connected to a computer, an LED array containing 240 LEDs (each LED provided red light with a wavelength of 660 nm and infrared LED light at a wavelength of 840 nm) and a laser probe which provided infrared light with a wavelength of 825 nm.^1^ We applied a single session of the LLLT protocol to target the lumbar and sacral area using parameters developed by Dr. Fred Kahn and colleagues (Kahn, 2022). Two arrays were used simultaneously at positions A and C for 10 min and then at positions B and D for 10 min. The array positions are shown in Figure 1A, and the LLLT protocol is given in Table 1. The LED arrays generated continuous red light for the first 5 min and infrared light pulses at 20 Hz for the next 5 min at each position. The arrays were followed by IR Laser Probe stimulations generating infrared laser light at 825 nm for 10 min. The laser probe contains one laser diode with a touch sensor to turn ON only upon touching the skin. Two laser probes were used simultaneously, one on each side of the spinal cord starting from the lumbar spine points L1-A as shown in Figure 1B for 10 s and then moving laterally on both sides by 1 cm marked as point L1-B and again stimulated for 10 s and in the third step, moving further away from the previous point by 1 cm marked as L1-C and stimulated for 10 s (Figure 1B). Similarly, for L2-L5 and S1-S5 to target the sacral sensory, sympathetic and parasympathetic nerves. Stimulating L1-S5 [three points lateral to the spinal cord for each (Figure 1B)] took 5 min using two probes simultaneously, the procedure then was repeated for the second time and the total stimulation time for the IR laser probe was 10 min. The placements of the laser probes are shown in Figure 1B. The technical specifications for LED array and the IR laser probe are given in Table 2.

**TABLE 1:** Low level laser therapy protocol.

Session	Head used	Position	Light	Pulse frequency	Duty cycle	Duration (min)
1	Baseline					6
2	LED arrays	A and C	Red, 660 nm	Continuous		5
			IR, 840 nm	20 Hz.	50%	5
3	LED arrays	B and D	Red, 660 nm	Continuous		5
			IR, 840 nm	20 Hz.	50%	5
4	Laser probes		IR, 825 nm	Continuous		10
5	Recovery					6

**TABLE 2:** Specifications for LED array and IR laser probe.

	LED array	IR Laser probe
Average power	1,000 mW	180 mW
Wavelength (λ)	660 nm (Red)/840 nm (IR)	825 nm
Spot size	100 cm^2^	0.1 cm^2^
Power density	10 mW/cm^2^	1,800 mW/cm^2^
Time of application	5 min	10 min
Total energy	30,000 J	10,800 J
Energy density	3 J/cm^2^	1,080 J/cm^2^

Energy density = (energy × time)/spot size; power density = power/spot size.

### Sham study

To rule out a placebo effect, the LLLT protocol was repeated twice in six healthy volunteers such that the arrays and probe were not turned ON during the first round while they were turned ON during the second round. The sham protocol included baseline (6 min) followed by simultaneously placed LED arrays at positions A and C (10 min) followed by simultaneous array placement at positions B and D (10 min) and in the next step, two IR laser probes were used to work simultaneously on both sides of the spinal column for 10 min with a 6 min recovery period (Figure 1).

### Visual representation of autonomic nervous system activity

The HRV spectrogram of the RR intervals signal was generated as an image for each step of the LLLT protocol. The ECG and impedance signals were imported into ImageJ using Cardio Images plugins.^2^ In the Cardio Images plugins, the peak detection and correction of the ECG signal was carried out by the Pan-Tomkins algorithm and a neural networks model generated and trained in TensorFlow with manual checking and editing of wrongly detected/edited R peaks. The tachogram of RR intervals was plotted as a raster image using a sampling frequency of 10 Hz, an image width of 5 s with cubic interpolation in the Intervals plugin. The Frequency Win plugin was used to calculate FFT spectra of the tachogram raster image using a window length of the 60 s and intervals of 10 s. The power spectra were collated into an image with time on the y-axis and frequency on the x-axis with pixel intensity as amplitude. The Win frequency plugin generated the HRV spectrogram from 0 to 5 Hz to study the RSA/HF (0.15–0.5 Hz.) band only, which represents the parasympathetic activity at normal breathing frequencies (Berntson et al., 1997). Lower frequency bands (0–0.15 Hz.), as well as the frequency band above 1 Hz, were removed in MATLAB. The spectrogram with the frequency band of 0.15–1 Hz was plotted as an image. The raster image of RR intervals was also read into MATLAB and was used to calculate RMSSD and SI, which were also plotted as aligned images. RMSSD and SI measured parasympathetic and sympathetic activity, respectively. These images were generated for the whole recording of the patients before the LLLT session—during array at AC—during array at BD—during IR laser probe—during recovery. The same procedure was used for the sham study.

### Statistical analysis

The HRV parameters (RSA, RMSSD, SI, SI/RSA, SI/RMSSD) and HR were calculated from the recorded ECG signal for each stage of the LLLT protocol, including baseline and recovery for each patient. The changes in each parameter during each stage were assessed statistically for 41 patients using GraphPad Prism software. The data were first checked for normality (gaussian distribution) using the Shapiro-Wilk Normality test. A dataset was normally distributed if all the columns (before-Array AC-Array BD-Probe-Recovery) passed the Shapiro-Wilk Normality test. In the case of the normally distributed data set One-Way-ANOVA followed by Holm-Sidak, multiple comparison tests were used to see the changes in the HRV parameter during each stage of the LLLT protocol. If data was not normally distributed, the Friedman test followed by Dunn’s multiple comparison test was applied to check the changes in that parameter. For subgroup analyses, the patients were divided into their main symptom: chronic constipation, fecal incontinence or both constipation and fecal incontinence. To identify the correlation between the major symptom with the HRV response during LLLT, a change in a HRV variable between baseline and each step of LLLT was assessed for correlation using the Pearson correlation test. A value of r greater than 0.50 or less than −0.50 with *p*-value of less than 0.05 was used as criteria for correlation. For the Sham protocol, two-way Anova followed by Bonferroni’s multiple comparison test was used to compare the changes in HRV parameters during the actual and sham protocol. The significance level was set at *p* < 0.05.

The data are presented as the effect of the light arrays, focusing on the outcome measured during activation of the BD array combination and during laser probe stimulation. The figures will show the data from each part of the stimulation protocol.

## Results

### Parasympathetic reactivity in response to light arrays and laser probe activation

**FIGURE 2:**
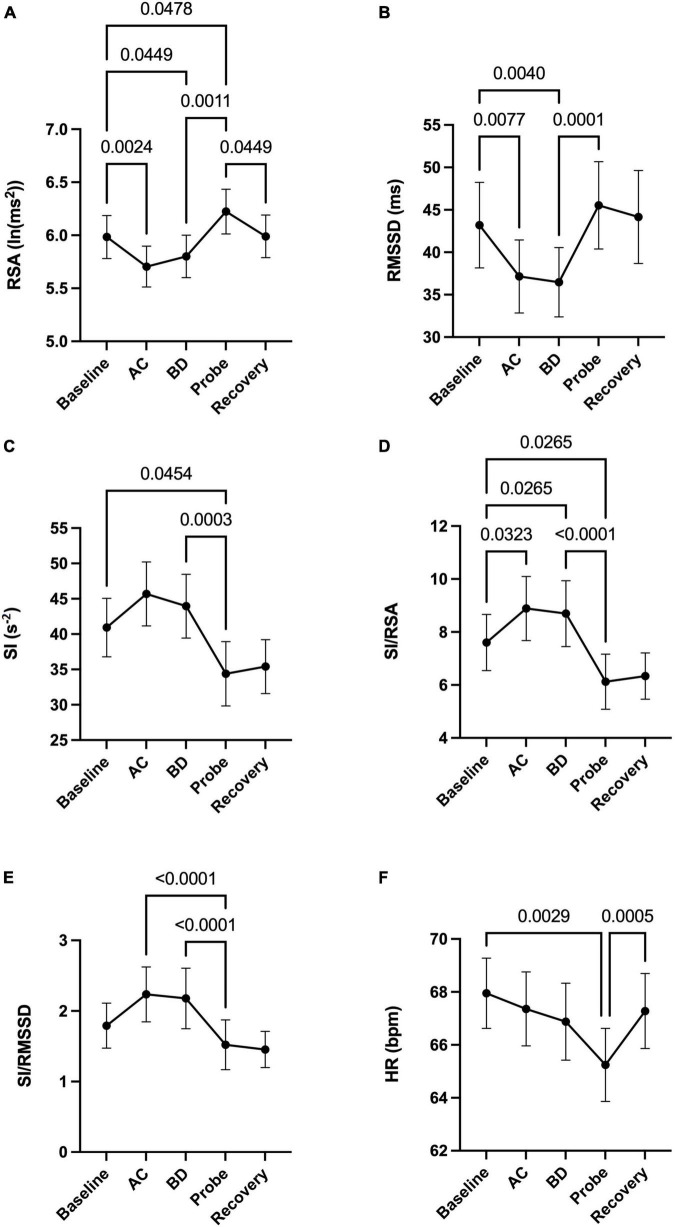
Autonomic nervous system modulation as deduced from HRV changes during one session of low-level laser therapy, stimulating the lumbar and sacral spine. **(A)** RSA, **(B)** RMSSD, **(C)** SI, **(D)** SI/RSA, **(E)** SI/RMSSD, **(F)** HR. Average values ± SEM from 41 patients with chronic colonic motility dysfunction.

Activation of the light arrays significantly decreases RSA from 5.98 to 5.80 ln (ms^2^) (Figure 2A), consistent with a significant decrease in RMSSD (Figure 2B).

Laser probe stimulation increased the RSA significantly to 6.22 ln (ms^2^) (Figure 2A). After the infrared laser probe stimulation, the RSA decreased back to 5.99 during recovery. Consistently, the RMSSD significantly increased in the period of laser probe stimulation.

**FIGURE 3:**
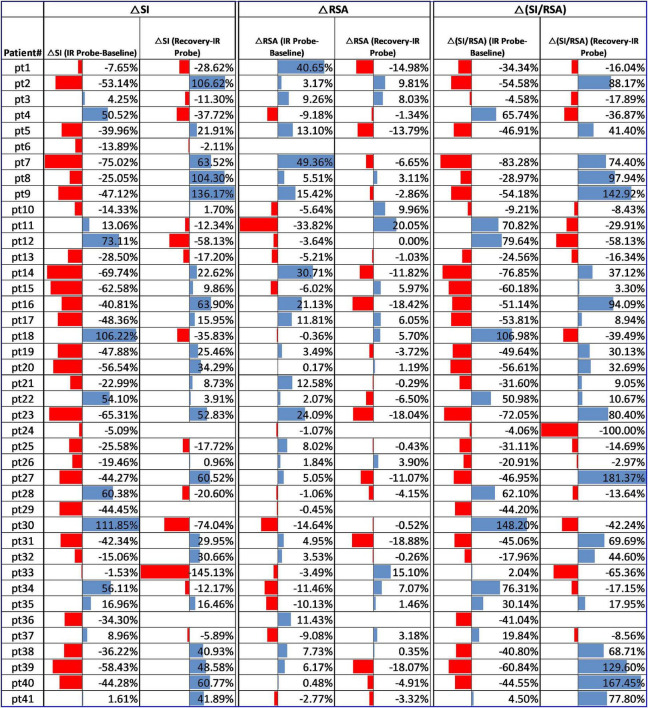
Change in RSA, SI, and SI/RSA due to laser probe stimulation and recovery in all 41 patients.

**FIGURE 4:**
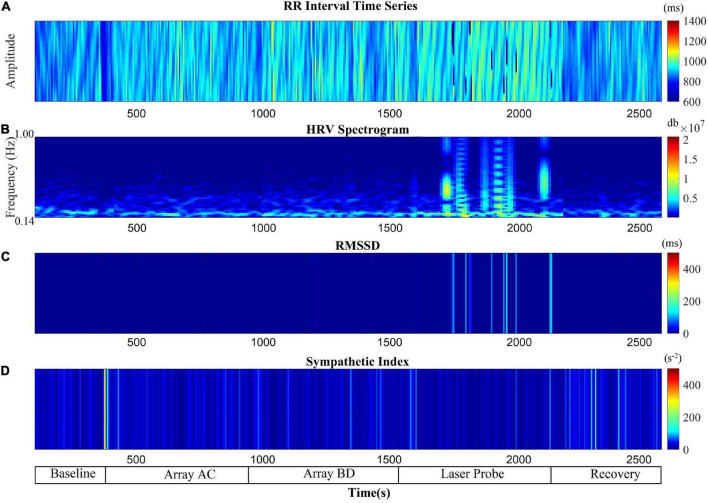
Autonomic nervous system modulation as deduced from HRV changes during the entire protocol of one session of low-level laser therapy in one patient. **(A)** RR Intervals time series, **(B)** HF band power, **(C)** RMSSD, **(D)** SI.

The RSA and RMSSD values at baseline and recovery were not significantly different (Figures 2A,B). The percentage change in RSA due to laser probe stimulation and its subsequent recovery for each patient are shown in Figure 3. Figure 4 shows a continuous assessment of the HRV parameters during the entire LLLT protocol in one patient. Figure 4B represents the HF power band from which the RSA is derived. A dramatic increase in parasympathetic activity occurs, measured by HF power and RMSSD during probe stimulation.

### Sympathetic reactivity in response to light arrays and laser probe activation

Laser probe stimulation decreased sympathetic activity without immediate recovery.

The sympathetic index (SI) measured the sympathetic nervous system response to the LLLT stimulations. The SI value numerically but not significantly increased during light array stimulation but significantly decreased from 41.3 to 34.8 s^–2^ during probe stimulation (Figure 2C). The SI values did not recover within 5 min following laser probe stimulation; stimulation decreased SI 17%, in the recovery period the decrease was still 12%. The percentage change in SI due to laser probe stimulation and the subsequent recovery values for each patient are shown in Figure 3.

### Assessing shifts in parasympathetic or sympathetic dominance

The ratios SI/RSA and SI/RMSSD showed a shift toward parasympathetic activity in response to the one-time LLLT session, dominated by a reduction in sympathetic activity.

During the laser probe stimulation, SI/RSA significantly decreased from a baseline value of 7.60 to 6.12, a 19.5% decrease. SI/RSA significantly decreased from the light arrays’ effect at 8.30 to 6.12, a 29.7% decrease (Figure 2D). The value of SI/RSA remained at 6.34 during the recovery period suggesting a sustained effect of the probe. SI/RMSSD showed numerically the same direction of change with a significant effect of the laser probe compared to the effect of the light arrays (Figure 2E).

Figure 3 shows the percentage change in SI/RSA during probe stimulation of all patients, indicating a shift toward parasympathetic activity in 30 out of the 41 patients.

The change in RSA and the change in SI, in response to LLLT were assessed for correlation with either constipation, fecal incontinence or the combination, but no correlation was observed, hence the changes in the HRV parameters were not specific for any of the symptom groups.

### Assessing heart rate changes during a one-time low-level laser therapy session

Comparing before and after the LLLT session, there was no significant change in heart rate. However, the average heart rate decreased by 2.7 beats per minute during laser probe stimulation compared to baseline. It recovered back to its baseline value after laser stimulation (Figure 2F).

### Sham neuromodulation on healthy subjects

Sham activation of light arrays and laser probe did not significantly change HRV parameters.

**FIGURE 5:**
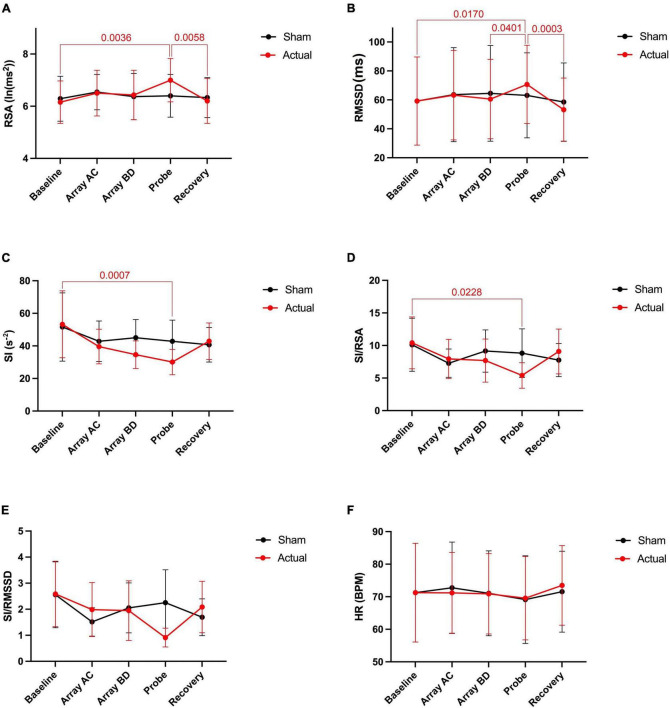
Autonomic nervous system modulation as deduced from HRV changes during one session of low-level laser therapy, stimulating the lumbar and sacral spine. **(A)** RSA, **(B)** RMSSD, **(C)** SI, **(D)** SI/RSA, **(E)** SI/RMSSD, **(F)** HR. Average values ± SEM from 6 healthy subjects during sham (placement of arrays and probe but no stimulation) and during actual activation of the arrays and probe.

In six healthy volunteers, laser probe stimulation increased RSA significantly from 6.16 during baseline to 7.00 ln (ms^2^); this decreased back to 6.20 ln (ms^2^) during recovery (Figure 5A). The sham procedure did not show any significant change in RSA during any step (Figure 5A). Similarly, the RMSSD increased significantly to 70.70 ms during laser probe stimulations compared to the baseline value of 59.19 ms (Figure 5B). RMSSD did not show any significant change during the sham protocol (Figure 5B).

The average value SI decreased significantly from 53.27 s^–2^ during baseline to 30.07 s^–2^ during laser probe stimulation (Figure 5C). The sham procedure did not show a significant change in SI during any step (Figure 5C).

During laser probe stimulation, the SI/RSA markedly decreased from its average baseline value of 10.41 to 5.41. This is almost a 50% decrease, which indicates a shift of autonomic dominance toward parasympathetic nervous system activity during laser probe stimulation. The sham procedure did not affect SI/RSA values during any step (Figure 5D). SI/RMSSD decreased numerically from 2.59 to 0.91 (Figure 5E). No change was observed during the sham procedure (Figure 5E). During actual and sham procedures, the heart rate did not change compared to baseline values (Figure 5F).

**FIGURE 6:**
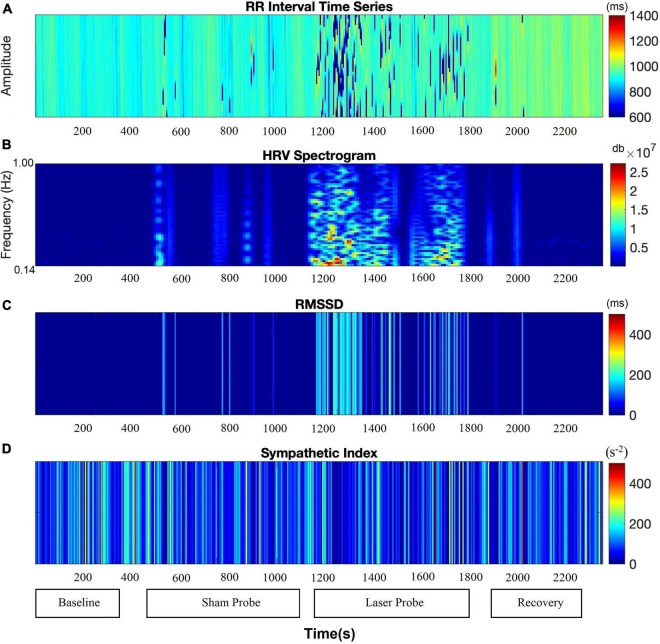
Comparison of autonomic nervous system activity during the application of the probe procedure with and without (sham) activating the probe. **(A)** RR Intervals time series, **(B)** HF band power, **(C)** RMSSD, **(D)** SI.

Figure 6 shows a continuous assessment of HRV parameters during the sham and laser probe stimulation of a single subject. The high-frequency band power of the RR interval signal indicates that during sham probe stimulation, there was no significant change in HF parasympathetic activity. In contrast, the amplitude of the HF power band increased markedly during actual laser probe stimulation compared to baseline, sham, and recovery (Figure 6B). This was also reflected in an increased RMSSD amplitude during actual laser probe stimulations (Figure 6C). A decrease in SI amplitude during the actual laser probe stimulation was observed (Figure 6D), compared to sham probe, baseline, and recovery.

## Discussion

The overall effect of a single LLLT session was increased parasympathetic activity and decreased sympathetic activity, resulting in a shifting of autonomic activity into the parasympathetic domain. The protocol involved stimulation using light arrays followed by a laser probe, with the arrays, on average, decreasing and the laser probe, on average, increasing parasympathetic activity. The sham study showed that the change in autonomic activity was due to activation by light and not due to touching or pressing the probes onto the skin.

The LLLT protocol used was developed over many years by the Bioflex clinic in Toronto (see text footnote 1) and we adopted the protocol that is used in this clinic for patients with lumbar spine problems. Light is focused on the nerves exiting and entering the spinal cord, including sensory nerves with its DRG. DRG, not being part of the central nervous system, are outside the vertebrae. The pedicles associated with the vertebrae create a foramen for the spinal nerves to enter or exit the intervertebral foramen. DRG can be close to the pedicles and can be obscured by the articular processes of the vertebrae, in particular related to the large lumbar vertebrae. Hence, the laser stimulation is focused on the areas immediately outside the body of the spinal cord.

Our focused objective was to investigate whether a single session of LLLT would demonstrably affect the autonomic nervous system as measured through HRV. The fact that HRV parameters significantly changed shows that the autonomic innervation to the heart was affected; hence a neural signal from the sacral-lumbar area evoked by low-level laser stimulation reached the brain stem. This will happen when nerve action potentials are evoked in the ascending autonomic nerves. The arrays and the probe gave different responses, likely because the probe light has a much higher intensity and will penetrate deeper in the tissue, potentially activating different neuronal circuitries.

Constipation is the inability of physiological stimuli such as eating or rectal filling to evoke effective defecation reflexes. Hence the extrinsic autonomic neural circuitries that facilitate defecation reflexes are not optimally functioning. The goal of LLLT concerning colonic motility disorders is to neuromodulate the circuitry of the autonomic nervous system so that normal reflexes are restored. Stimulation with LLLT may stimulate neurons to fire action potentials with, as a consequence, the strengthening of the functionality of the synapses while also stimulating increased energy production and nutrient delivery through cellular activation and vasodilation (Chung et al., 2012; Rola et al., 2014). This would allow for recovery of the functionality of neuronal circuitries and neural regeneration (Chen et al., 2014) and ultimately help in the restoration of the defecation reflex (Furness et al., 2014; Milkova et al., 2020). The data of the present study do not inform about the likelihood of success of this therapy but give credence to exploring sacral neuromodulation using LLLT as a treatment for chronic colonic motility dysfunction.

## Data availability statement

The original contributions presented in the study are included in the article/supplementary material, further inquiries can be directed to the corresponding author/s.

## Ethics statement

The studies involving human participants were reviewed and approved by the Hamilton Integrated Research Ethics Board. The patients/participants provided their written informed consent to participate in this study.

## Author contributions

MA analyzed all the data, contributed to interpretation, and wrote a manuscript draft. SS made a significant contribution to data analysis and data interpretation at the beginning of the project. NM and LL contributed to patient assessment and data analysis. KS made a substantial contribution to data analysis. JH and J-HC designed the study and contributed to data collection, data analysis, interpretation, and revisions to the manuscript. All authors contributed to the article and approved the submitted version.

## Footnotes

1
https://bioflexlaser.com

2
http://www.scepticalphysiologist.com

## Abbreviations

LLLT: Low-Level Laser Therapy
HRV: Heart rate Variability
RSA: Respiratory Sinus Arrhythmia
SI: Baevsky’s Stress Index or Sympathetic Index
RMSSD: Root Mean Square of Successive Differences.
